# Method for Controlling Electrical Properties of Single-Layer Graphene Nanoribbons via Adsorbed Planar Molecular Nanoparticles

**DOI:** 10.1038/srep12341

**Published:** 2015-07-24

**Authors:** Hirofumi Tanaka, Ryo Arima, Minoru Fukumori, Daisuke Tanaka, Ryota Negishi, Yoshihiro Kobayashi, Seiya Kasai, Toyo Kazu Yamada, Takuji Ogawa

**Affiliations:** 1 Graduate School of Science, Osaka University, 1-1 Machikaneyama, Toyonaka, Osaka 560-0043, Japan; 2 Graduate School of Life Science and Systems Engineering, Kyushu Institute of Technology (Kyutech), 2-1 Hibikino, Wakamatsu, Kitakyushu 808-0196, Japan; 3 Graduate School of Engineering, Osaka University, 2-1 Yamadaoka, Suita, Osaka 565-0871, Japan; 4 Research Center for Integrated Quantum Electronics, Hokkaido University, North 13, West 8, Sapporo 060-8628, Japan; 5 Graduate School of Advanced Integration Science, Chiba University, 1-33, Yayoi, Inage, Chiba 263-8522, Japan

## Abstract

A simple method for fabricating single-layer graphene nanoribbons (sGNRs) from double-walled carbon nanotubes (DWNTs) was developed. A sonication treatment was employed to unzip the DWNTs by inducing defects in them through annealing at 500 °C. The unzipped DWNTs yielded double-layered GNRs (dGNRs). Further sonication allowed each dGNR to be unpeeled into two sGNRs. Purification performed using a high-speed centrifuge ensured that more than 99% of the formed GNRs were sGNRs. The changes induced in the electrical properties of the obtained sGNR by the absorption of nanoparticles of planar molecule, naphthalenediimide (NDI), were investigated. The shape of the *I-V* curve of the sGNRs varied with the number of NDI nanoparticles adsorbed. This was suggestive of the existence of a band gap at the narrow-necked part near the NDI-adsorbing area of the sGNRs.

Ever since remarkable two-dimensional electron system of graphene was discovered^1^, it has come to be used in a variety of applications. Because graphene’s carrier mobility is extremely high (2 × 10^5^ cm^2^ V^−1^ s^−1^)^2^,^3^, it can be used as a silicon substitute in transistors that have to operate at high frequencies. Large-area graphene sheets have been used to successfully fabricate circuits on SiC/Si wafers^4^. On the other hand, as scaled-down graphene-based devices and wiring are developed, it has become important to investigate the electrical properties of graphene nanoribbons (GNRs), particularly single-layer GNRs (sGNRs). Several methods of fabricating GNRs have been reported, including lithographic^5^,^6^,^7^ and chemical^8^ processes. A popular method of synthesizing GNRs in large amounts is to unzip carbon nanotubes (CNTs) directly in solution^9^,^10^,^11^,^12^,^13^. The synthesis of sGNR by this technique involves first obtaining multilayered GNRs (mGNRs) by unzipping multiwalled carbon NTs (MWNTs, number of layers ≥ 3) in solution^14^ and then unpeeling one of the layers of the mGNRs. Although we have also been able to successfully obtain sGNRs directly by unzipping SWNTs in solution, we intend to report these results elsewhere. This is because, in the present study, we focused on the changes induced in the electrical properties of sGNRs by the adsorption of organic nanoparticles onto them, given that the sGNRs exhibited semiconductor-like characteristics because their width was less than 10 nm. In this study, we were able to unzip double-walled carbon nanotubes (DWNTs) instead of MWNTs to obtain semimetallic sGNRs with a width of 15–50 nm.

In this study, our aim was to devise a method for obtaining sGNRs in high yields. DWNTs were used as the starting material instead of MWNTs, in contrast to what has been done in previous studies. We found that unzipping DWNTs yielded double-layered GNRs (dGNRs), which were easier to unpeel and separate into two individual sGNRs by further sonication.

The second aim of the study was to investigate how the electrical properties of sGNRs are affected when nanoparticles of planar molecules are adsorbed onto them. Controlling the electrical properties of sGNRs, including their band gap, is also important for the development of graphene-based electronics. Only a few methods for controlling the band structure of graphene have been reported. In one method, the band structure of graphene is controlled by forming a nanomesh by fabricating periodic holes (called “necks”) of varying sizes at different distances^15^. Although this method is a breakthrough in the development of graphene-based electronics, the structures of the devices based on such nanomeshes would still be very complex, and the devices time consuming to fabricate. Developing easier methods of controlling the electrical properties of graphene is therefore essential.

We attempted to achieve this goal by modulating the adsorption of nanoparticles of organic molecules onto graphene. We have already reported the effect of adsorbed organic nanoparticles on the electrical properties of individual SWNTs. When nanoparticles of 5,15-bispentylporphyrinato zinc(II) (porphyrin) is adsorbed on an SWNT, conduction through the nanoparticles is followed by Fowler-Nordheim tunnelling^16^. On the other hand, if N,N’-bis(*n*-alkyl)tetracarbonatenaphthalenediimide (C*x*-NDI, where *x* is the number of methylene units in the alkyl side chains) is adsorbed, conduction is followed by Schottky emission^17^. These differences are attributable to the differences in the highest occupied molecular orbital (HOMO) and lowest unoccupied molecular orbital (LUMO) levels of the porphyrin (donor) and C*x*-NDI (acceptor) molecules. Further, we also determined the *I-V* characteristics of the graphene samples, since we were also interested in elucidating the changes induced in the electrical properties of graphene by these particles. It was found that the electrical properties of graphene could be controlled merely by controlling the number of nanoparticles adsorbed.

## Experimental

**Figure 1:**
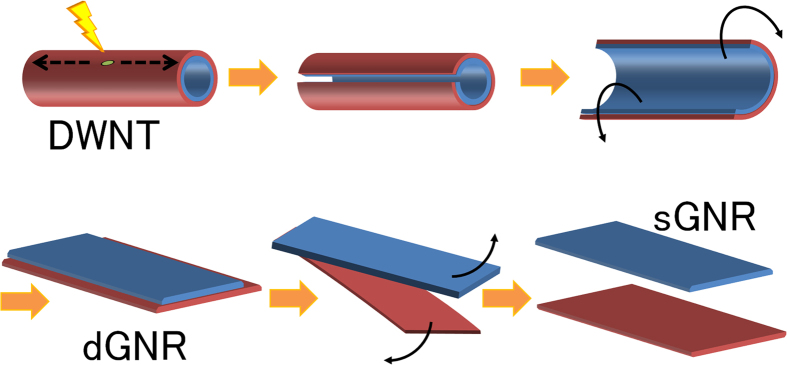
Procedure for obtaining single-layered graphene nanoribbons (sGNRs) from double-walled carbon nanotubes (DWNTs). (**a**) A defect is created in a DWNT by annealing. (**b**) Sonicating the DWNT allows it to be unzipped, starting at the defect. (**c**) The DWNT is unzipped completely and (**d**) a flat, double-layered GNR (dGNR) is obtained. (**e**) Further sonication splits the dGNR into two individual sGNRs, and (**f**) the sGNRs can be separated by centrifugation. The sGNR obtained from the outer layer of the DWNT (red) is slightly wider than that obtained from the inner layer (blue), owing to the geometry of the DWNT.

We obtained the dGNRs as well as the sGNRs via sonication treatments performed using a previously reported procedure^11^. DWNTs having diameters of 3–15 nm and synthesised by catalyst-based chemical vapour deposition (CVD) were obtained commercially (Tokyo Ohka Kogyo Co., Ltd.). These were used as the starting material instead of MWNTs. The purchased DWNTs were annealed in air at 500 °C to induce defects in them. They were then dispersed in an organic solution of 7.5 mg of poly(*m*-phenylenevinylene-co-2,5-dioctoxy-*p*-phenylenevinylene) (PmPV) in 20 ml of 1,2-dichloroethane. Under sonication performed at 37 kHz and 600 W, the annealed DWNTs could be unzipped into high-quality dGNRs. Further, by increasing the sonication time from 0.5 h to 16 h while maintaining the power at the same level, the dGNRs could be unpeeled to form sGNRs. The solution was then ultracentrifuged at 45620 G (20000 rpm; TOMY Suprema21 High Speed Centrifuge) for 2 h to remove any nanotubes that remained unzipped, as well as the amorphous carbon-like impurities present in the starting material. The supernatant was then cast onto a cleaved mica or SiO_2_ substrate, and the substrate was annealed at 350 °C for 1 h in air to remove all of the PmPV, yielding dispersed sGNRs. Raman spectroscopy was performed on the sGNRs using a RENISHAW inVia Reflex/Stream Line system and a Horiba HR800 system with 633 and 785 nm excitation lasers. The electrical properties of the sGNRs were measured using point-contact current-imaging atomic force microscopy (PCI-AFM)^18^,^19^ and a three-electrode field-effect transistor (FET) structure. The unzipping process described above is illustrated in Fig. 1.

Next, a toluene solution of the organic molecule C15-NDI was cast on the substrate precast with the sGNRs as per a previously reported procedure^19^. During the evaporation of the solvent, self-assembled nanoparticles of C15-NDI were formed on the sGNRs through π-π stacking. The electrical characteristics of the complex were also measured by PCI-AFM and a three-electrode FET structure with a back gate using a four-probe system (Lakeshore, ProbeStation TTP). Electrodes with a Ni(5 nm)/Au(30 nm) structure were fabricated through ultraviolet (UV) lithography. The measurements were controlled with an Advantest R6245 two-channel *I-V* source monitor, which was interfaced with a computer through a GPIB-SCSI board as per the NI-488.2 protocol. The data were collected using a self-devised procedure and the Igor Pro 4.0 (WaveMetrics) software.

## Results and Discussion

**Figure 2:**
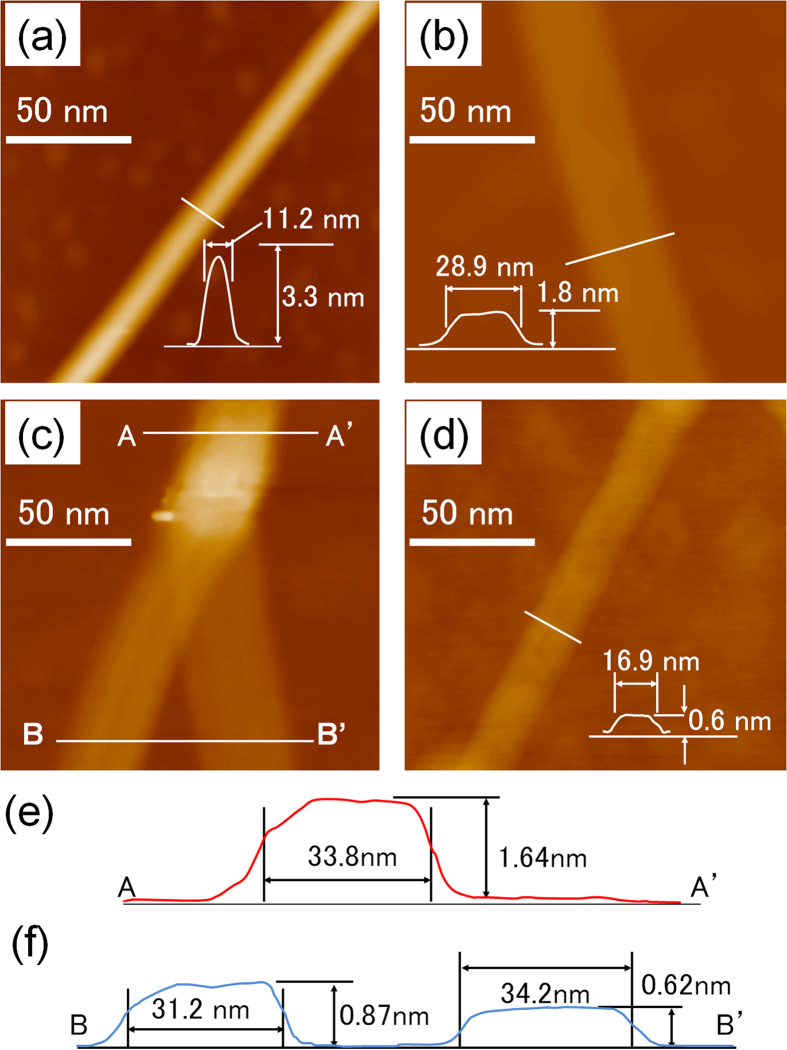
AFM images of (**a**) a DWNT used as the starting material, (**b**) a dGNR obtained by the unzipping of the DWNT, (**c**) the region at which the dGNR splits into two sGNRs, (**d**) a resulting sGNR. Each scale bar is 50 nm. (**e**) Line profile of the dGNR in (**c**), obtained along the line A-A′. The height and width of the dGNR are 1.64 nm and 33.8 nm, respectively. (**e**) Line profile of the dGNR in (**c**), obtained along the line B-B′. The height and width of the sGNR on the left are 0.87 nm and 31.2 nm, respectively, and those of the sGNR on the right are 0.62 nm and 34.2 nm, respectively. The insets in (**a**,**b**,**d**) show the height profile along the white line in each case. The images (**a**–**d**) correspond to different samples.

The AFM images of the DWNTs used as the starting material showed that they had diameters of 3–15 nm and lengths greater than 1000 nm (Fig. 2(a)). Annealing at 500 °C and the subsequent sonication allowed the DWNTs to be unzipped, resulting in dGNRs (Fig. 2(b)). No SWNTs resulting from the unzipping of only the outer walls of the DWNTs were observed. This suggested that all the unzipped DWNTs formed dGNRs. The dGNRs had a height of 1.6 nm, a value similar to that reported previously^13^. The observed thickness of the dGNRs was slightly higher than the theoretical value, owing to the roughness of the SiO_2_ substrate and the defects induced in the dGNRs by sp3 bonding. Figure 2(c) shows an AFM image of the Y-shaped point at which a dGNR splits or unpeels into two sGNRs; the GNRs corresponding to this stage of the unpeeling process are called yGNRs, in order to distinguish them from the dGNRs and sGNRs. The height and width of the yGNRs along the AA′ line (double-layered area) were 1.64 nm and 33.8 nm, respectively (see Fig. 2(b)). Similarly, those along the BB’ line (single-layered area) were 0.87 nm and 31.2 nm, respectively, those for the sGNR on the left and 0.62 nm and 34.2 nm, respectively, for the sGNR on the right (see Fig. 2(c,e,f)). These values were determined from the AFM profiles of the GNRs. Although the observed widths were greater than the actual values, owing to the curvature of the AFM tip, the difference in the widths could be compared in the same image. The difference in the width owing to blurriness caused by the roundness of the cantilever tip can be expected to be less than 0.5 nm in this range, if the same cantilever is used^20^. Figure 2(c) shows which sGNR was formed from the inner wall of the DWNT and which one was obtained from the outer wall. The sGNR on the left is 3 nm narrower than the one on the right. Since the ribbons formed from the inner walls of the DWNTs would be narrower, it can be concluded that the sGNR on the left in the figure is formed from an inner wall and the one on the right is obtained from an outer wall. Further, the reason the sGNR on the left looks thicker than the one on the right is that the former was formed from the upper layer of the dGNR and was not touching the surface of the substrate. A difference in the widths of approximately 3 nm corresponds to a difference of 1 nm in the outer and inner diameters of the DWNTs. Finally, the Raman spectra confirmed that sGNRs were obtained in high yields using the unzipping process with no existence of DWNT residue because no peaks attributable to the radial breathing mode were observed (Fig. S3).

Thus, it was confirmed that DWNTs are a better starting material than MWNTs for obtaining semi-metallic sGNRs in high yields. The spring constant of DWNTs is considered higher than that of MWNTs. This is because the smaller radius of DWNTs results in the bond angle between neighbouring carbon atoms being higher. This suggests that a greater amount of energy is stored in the case of DWNTs. This energy can be used for the unzipping of the CNTs along their long axis. Although we found that SWNTs are also suitable for producing narrower sGNRs, we will report these results elsewhere, because we wished to focus on opening the band gap of the semimetallic GNRs (wider than 10 nm) in the present work. The *I-V* characteristics of the sGNRs and dGNRs obtained from DWNTs were determined (Fig. S4). Both types of GNRs exhibited semimetallic properties.

**Figure 3:**
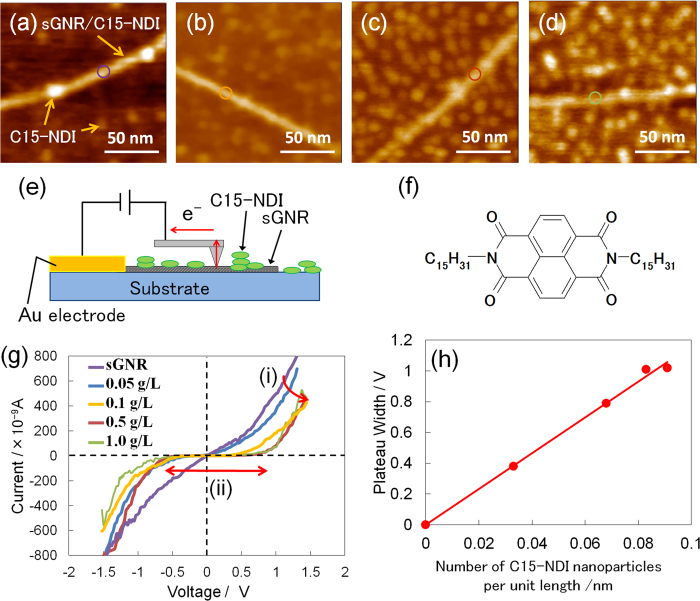
(**a**–**d**) AFM images of sGNRs after the adsorption of molecular nanoparticles of NDI onto them by the drop casting of C15-NDI solutions of different concentrations. The concentrations of the solutions were (**a**) 0.05 g L^−1^, (**b**) 0.1 g L^−1^, (**c**) 0.5 g L^−1^, and (**d**) 1.0 g L^−1^. (**e**) Schematic of the setup used to determine the *I*-*V* characteristics of the NDI-adsorbing sGNRs using PCI-AFM. (**f**) Molecular structure of C15-NDI. (**g**) *I*-*V* curves of the C15-NDI/GNR complex for different concentrations of the C15-NDI solution. As the concentration of the solution was increased, (i) the current decreased gradually and (ii) the plateau width (PW) increased. (**h**) The values of the PW plotted as a function of the number of C15-NDI nanoparticles adsorbed on the sGNRs per unit length of the sGNRs. Owing to steric hindrance, the upper limit for the number of nanoparticles adsorbed per unit length was approximately 0.1.

**Figure 4:**
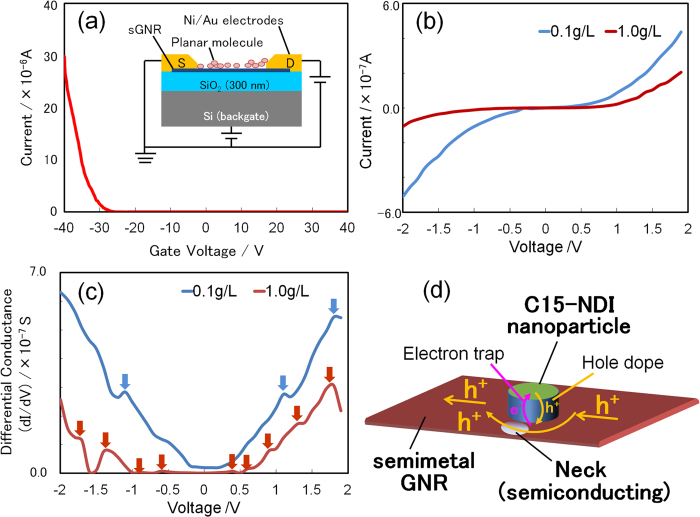
Results obtained using the FET structure. (**a**) Gate property of the FET structure based on the C15-NDI/GNR complex. The sGNRs exhibited p-type semiconductor behavior after the adsorption of the NDI nanoparticles. The inset shows the back-gated FET structure employed for the measurements. (**b**) *I*-*V* curves of the C15-NDI/GNR complex at Vg = 0, obtained for C15-NDI solution concentrations of 0.1 g L^−1^ and 1.0 g L^−1^. (**c**) Differential conductance values obtained from (**b**). Three peaks are observed in the curve corresponding to the 0.1 g L^−1^ solution and nine peaks in one corresponding to the 1.0 g L^−1^ solution. These peaks indicate the points at which Zener-like tunnelling occurred, strongly implying the existence of a band gap in the NDI-adsorbing sGNR. (**d**) Schematic showing the electron path in an NDI-adsorbing sGNR. The NDI nanoparticle becomes an electron-trapping site, and current flows through the necks of the sGNR.

Next, the solution of C15-NDI was drop cast onto the Si substrate. As a result, the C15-NDI molecules, which are planar, were adsorbed as nanoparticles on the sGNRs. The *I-V* characteristics of the sGNRs were measured again. Figure 3(a–d) show AFM images of sGNRs with C15-NDI nanoparticles adsorbed on their surfaces by the drop casting of solutions of various concentrations. By increasing the concentration of the C15-NDI solution, the number of nanoparticles adsorbed on the substrate surface as well as on the sGNRs increased. The *I-V* curves of the GNR/C15-NDI complex (Fig.3 (f)) were determined by PCI-AFM (Fig. 3(e)) for different concentrations (Fig. 3(g)). The part of the *I-V* curve corresponding to d*I*/d*V* = 0 represents the plateau width (PW) of the complex. As the concentration of the C15-NDI solution was increased, the current decreased gradually while the PW increased. Figure 3(h) shows the PW values plotted against the number of nanoparticles adsorbed per unit length of an sGNR. The value of PW varied in proportion to the number of adsorbed nanoparticles. It was also found that the number of adsorbed nanoparticles reached a maximum when the concentration of the solution was approximately 1.0 g L^−1^. The collected data included information about the band structure of the sGNRs, because the sGNRs transformed into a *p*-type semiconductor material; this was determined from the FET-based measurements (Fig. 4(a)), even though the PW values are not directly indicative of the band gap itself. The reason the PW value changed with the number of adsorbed nanoparticles is the following: the positions where the nanoparticles are adsorbed become localised electron-trapping sites. As the number of molecules adsorbed on a GNR is approximately 10 times larger than the number of conduction electrons, the electrons trapped by the nanoparticles no longer contribute to conduction, and hole conduction mainly occurs in the other areas of the sGNR. This phenomenon is similar to conduction through the “necks” in a graphene nanomesh^15^. The conduction path must become narrower as the size of the adsorbed nanoparticles increases. It has also been reported that, if the effective width of sGNRs is low, their band gap is similar to that of a semiconductor^21^. This is probably the primary reason why sGNRs transformed into a semiconductor material from a semimetallic one, when the C15-NDI nanoparticles were adsorbed onto their surfaces. To determine whether this was indeed the case, we analysed the *I-V* curves obtained using a back-gated FET structure (inset of Fig. 4(a)) and a four-probe system. Figure 4(b) shows the *I-V* curves obtained using FET structures fabricated from the GNR/C15-NDI complexes synthesised using C15-NDI solutions with concentrations of 0.1 g L^−1^ and 1.0 g L^−1^. The differential conductance (d*I*/d*V-V*) curves derived from these *I-V* curves are shown in Fig. 4(c). The curve corresponding to the 0.1 g L^−1^ solution exhibits three peaks, while that corresponding to the 1.0 g L^−1^ solution contains nine peaks. The peaks are indicated by arrows and are evidence of the sudden changes in the current (called kinks) during the measurements.

It was assumed that the appearance of these kinks proved that the band gap of the sGNRs opened up around the neck structures, owing to the adsorption of the C15-NDI nanoparticles. This was because of the following reason: there are a number of high-resistance points (the previously mentioned necks) in the NDI-adsorbing sGNRs, because the holes are screened in very short distance of the neck along the long axis of the sGNRs. In such cases, when a bias is applied to the entire GNR, only a partial voltage is applied to the high-resistance regions of the necks. Every high-resistance neck forms a high-electron-field domain, giving rise to Zener-like tunnelling. When this type of tunnelling occurs, the current flowing through the sGNR increases suddenly, giving rise to the kinks noticed in the *I-V* curves, because the band gap is small enough to break down. Thus, the breakdown of current introduces the peaks observed in the d*I*/d*V-V* curves. To put it simply, the number of kinks in the *I-V* curve of a system equals the number of times tunnelling occurs in the system. Further, the number of times tunnelling occurs is equal to the number of semiconducting necks in an NDI-adsorbing sGNR. This proves that the high-resistance regions of an NDI-adsorbing sGNR break down at high voltages, even though this does not directly prove the existence of a band gap. In other words, the occurrence of tunnelling strongly implies than an NDI-adsorbing sGNR has a band gap. Thus, we can conclude that the band gap of an sGNR can be controlled by controlling the number of C15-NDI nanoparticles that are adsorbed onto it. This, in turn, can be varied by controlling the number of necks. This hypothesis is strongly supported by the FET data shown in Fig. 4(a), which is indicative of *p*-type semiconductor behavior.

The technique used in the present work to control the electrical properties, especially the opening band gap, of an sGNR by controlling the number of C15-NDI nanoparticles adsorbed onto it is a promising one and should aid in the fabrication of graphene-based nanodevices.

## Conclusion

It was shown that DWNTs are a much better starting material than MWNTs for unzipping and forming sGNRs. The unzipping of DWNTs yielded double-layered GNRs, which could be peeled to form single-layered GNRs. The electrical properties of the sGNRs could be controlled by varying the number of C15-NDI nanoparticles adsorbed onto them, making the technique a promising approach for band-gap engineering. The band gap of the sGNRs was attributable to the necking structures formed around the adsorbed C15-NDI nanoparticles. This technique should also be useful for controlling the functionality of conventional semiconductor devices. The plateau widths of the *I-V* curves of the NDI-adsorbing sGNRs varied in proportion to the number of adsorbed C15-NDI particles. Further, the electrical properties of the sGNRs also changed, with the sGNRs going from being semimetallic in nature to a p-type semiconductor, after the adsorption of the molecular C15-NDI nanoparticles.

## Additional Information

**How to cite this article**: Tanaka, H. *et al.* Method for Controlling Electrical Properties of Single-Layer Graphene Nanoribbons via Adsorbed Planar Molecular Nanoparticles. *Sci. Rep.* **5**, 12341; doi: 10.1038/srep12341 (2015).

## Supplementary Material

Supplementary Information

## References

[b1] Novoselov K. S. *et al.* Electric field effect in atomically thin carbon films. Science 306, 666–669 (2004). 15499015 10.1126/science.1102896

[b2] Orlita M. *et al.* Approaching the Dirac Point in High-Mobility Multilayer Epitaxial Graphene. Phys. Rev. Lett. 101, (2008). 10.1103/PhysRevLett.101.267601 19437673

[b3] Bolotin K. I. *et al.* Ultrahigh electron mobility in suspended graphene. Solid State Commun. 146, 351–355 (2008).

[b4] Lin Y. M. *et al.* 100-GHz Transistors from Wafer-Scale Epitaxial Graphene. Science 327, 662–662 (2010). 20133565 10.1126/science.1184289

[b5] Chen Z., Lin Y. M., Rooks M. J. & Avouris P. Graphene nano-ribbon electronics. Physica E-Low-Dimensional Systems & Nanostructures 40, 228–232 (2007).

[b6] Han M. Y., Ozyilmaz B., Zhang Y. B. & Kim P. Energy band-gap engineering of graphene nanoribbons. Phys. Rev. Lett. 98, 206805 (2007). 17677729 10.1103/PhysRevLett.98.206805

[b7] Tapaszto L., Dobrik G., Lambin P. & Biro L. P. Tailoring the atomic structure of graphene nanoribbons by scanning tunnelling microscope lithography. Nat. Nanotechnol. 3, 397–401 (2008). 18654562 10.1038/nnano.2008.149

[b8] Campos-Delgado J. *et al.* Bulk production of a new form of sp(2) carbon: Crystalline graphene nanoribbons. Nano Lett. 8, 2773–2778 (2008). 18700805 10.1021/nl801316d

[b9] Terrones M. Nanotubes Unzipped. Nature 458, 845–846 (2009). 19370025 10.1038/458845a

[b10] Kosynkin D. K. & D V. *et al.* Longitudinal unzipping of carbon nanotubes to form graphene nanoribbons. Nature 458, 872–877 (2009). 19370030 10.1038/nature07872

[b11] Jiao L. J. & L Y. *et al.* Facile synthesis of high-quality graphene nanoribbons. Nat. Nanotechnol. 5, 321–325 (2010). 20364133 10.1038/nnano.2010.54

[b12] Jiao L. J. & L Y. *et al.* Narrow graphene nanoribbons from carbon nanotubes. Nature 458, 877–880 (2009). 19370031 10.1038/nature07919

[b13] Shinde D. B. *et al.* Electrochemical unzipping of multi-walled carbon nanotubes for facile synthesis of high-quality graphene nanoribbons. J. Am. Chem. Soc. 133, 4168 (2011). 21388198 10.1021/ja1101739

[b14] Tao C. G. *et al.* Spatially resolving edge states of chiral graphene nanoribbons. Nat. Phys. 7, 616–620 (2011).

[b15] Bai J. W. *et al.* Graphene nanomesh. Nat. Nanotechnol. 5, 190–194 (2010). 20154685 10.1038/nnano.2010.8 PMC2901100

[b16] Tanaka H., Yajima T., Kawao M. & Ogawa T. Electronic properties of a single-walled carbon nanotube/150mer-porphyrin system measured by point-contact current imaging atomic force microscopy. J. Nanosci. Nanotechnol. 6, 1644–1648 (2006). 17025064 10.1166/jnn.2006.246

[b17] Tanaka H. *et al.* Influence of nanoparticle size to the electrical properties of naphthalenediimide on single-walled carbon nanotube wiring. Nanotechnology 23, 215701 (2012). 22551735 10.1088/0957-4484/23/21/215701

[b18] Otsuka Y., Naitoh Y., Matsumoto T. & Kawai T. Point-contact current-imaging atomic force microscopy: Measurement of contact resistance between single-walled carbon nanotubes in a bundle. Appl. Phys. Lett. 82, 1944–1946 (2003).

[b19] Tanaka H. *et al.* Porphyrin molecular nanodevices wired using single-walled carbon nanotubes. Adv. Mater. 18, 1411–1415 (2006).

[b20] Sikora A. Correction of structure width measurements performed with a combined shear-force/ tunnelling microscope. Measurement Scinence and Technology 18, 456–461 (2007).

[b21] Shimizu T. *et al.* Large intrinsic energy bandgaps in annealed nanotube-derived graphene nanoribbons. Nat. Nanotechnol. 6, 45–50 (2011). 21170040 10.1038/nnano.2010.249

