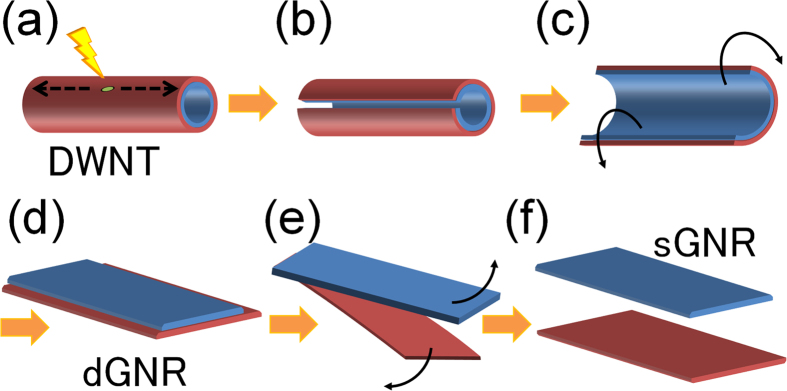# Corrigendum: Method for Controlling Electrical Properties of Single-Layer Graphene Nanoribbons via Adsorbed Planar Molecular Nanoparticles

**DOI:** 10.1038/srep14399

**Published:** 2015-10-20

**Authors:** Hirofumi Tanaka, Ryo Arima, Minoru Fukumori, Daisuke Tanaka, Ryota Negishi, Yoshihiro Kobayashi, Seiya Kasai, Toyo Kazu Yamada, Takuji Ogawa

Scientific Reports 5: Article number: 12341; 10.1038/srep12341 published online: 07242015; updated: 10202015.

In this Article, the Figure labels were omitted from Figure 1. The correct Figure 1 appears below.

In addition, there is a typographical error in the Acknowledgements section.

“HT also thanks Prof. S. Qiao of Fudan University, China, and Prof. K. F. Kelly of Rice University, USA, for fruitful discussions and Profs. K. Maeda and Y. Horibe of Kyutech, Japan, for support with the experiments.”

should read:

“HT also thanks Prof. S. Qiao of Fudan University, China, and Prof. K. F. Kelly of Rice University, USA, for fruitful discussions and Profs. T. Maeda and Y. Horibe of Kyutech, Japan, for support with the experiments.”

## Figures and Tables

**Figure 1:**