# Supplementary material for: Method for Controlling Electrical Properties of Single-Layer Graphene Nanoribbons via Adsorbed Planar Molecular Nanoparticles
Source: Sci Rep. 2015 Jul 24;5:12341. doi: 10.1038/srep12341 (PMC4513291; doi:10.1038/srep12341)
Supplement: Supplementary Information [file srep12341-s1.pdf]

Supplementary Information

Method for Controlling Electrical Properties of Single-Layer Graphene Nanoribbons via Adsorbed Planar Molecular Nanoparticles

Hirofumi Tanaka,[1,2,*] Ryo Arima,[1] Minoru Fukumori,[1] Daisuke Tanaka,[1,†] Ryota Negishi,[3] Yoshihiro Kobayashi,[3] Seiya Kasai,[4] Toyo-Kazu Yamada[5] and Takuji Ogawa[1]

[1]Graduate School of Science, Osaka University, 1-1 Machikaneyama, Toyonaka, Osaka 560-0043, Japan.

[2]Graduate School of Life Science and Systems Engineering, Kyushu Institute of Technology (Kyutech), 2-1 Hibikino, Wakamatsu, Kitakyushu 808-0196, Japan.

[3]Graduate School of Engineering, Osaka University, 2-1 Yamadaoka, Suita, Osaka 565-0871, Japan.

[4]Graduate School of Information Science and Technology, Hokkaido University, North 14, West 9, Kita, Sapporo 060-0814, Japan.

[5]Graduate School of Advanced Integration Science, Chiba University, 1-33, Yayoi, Inage, Chiba 263-8522, Japan.

[†]Present address: Kwansei Gakuin University, 669-1337 Hyogo, Japan.

*Corresponding Author: tanaka@brain.kyutech.ac.jp

Figure S1 shows the material fractions of the DWNTs as the starting material and those of the dGNRs, yGNRs, and sGNRs as functions of the sonication time. The fractions were calculated from the AFM images such as the one shown in Fig. S2. After sonication for 8 h, almost all the DWNTs had disappeared and the fraction of the sGNR was greater than 90%. After sonication for 16 h, the fraction of sGNR was nearly 100%. Further, yGNRs, which formed because of the splitting of the dGNRs into two sGNRs and had a Y-shaped point and a length of more than 10 μm, were present in a fraction of less than 10%. After sonication for 8 h or longer, numerous defects were induced in the sGNRs, and their length decreased. Thus, a sonication time of 4 h was found to be optimal for obtaining high-quality sGNRs. The fractions were calculated by counting more than 100 GNRs in the AFM images. The DWNTs and dGNRs disappeared after sonication for 2 h and 4 h, respectively. Thus, it can be concluded that it is difficult to split the dGNRs into sGNRs perfectly if the yGNRs are long. After sonication for 0.5 h and 1 h, dGNRs and yGNRs, respectively, were present in the greatest fractions. These results support the conclusion that the unzipping process takes place via the steps shown in Fig. 1.

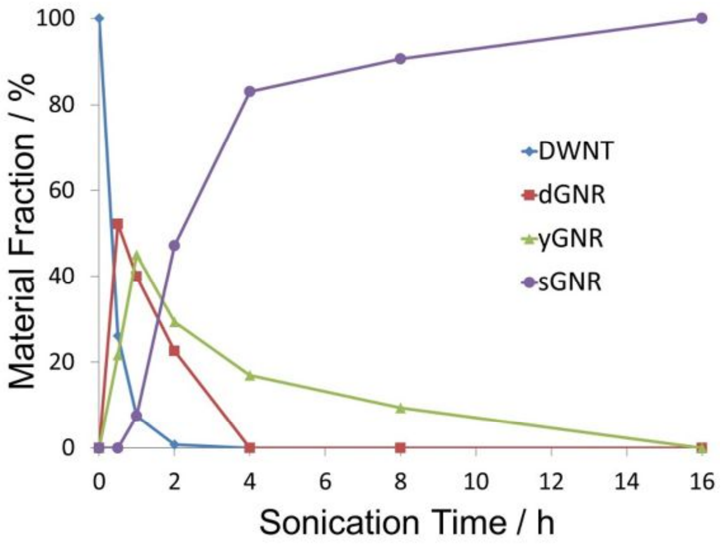

Fig. S1. Material fractions of various types of GNRs as functions of the sonication time. DWNTs were used as the starting material. The yGNRs were formed when a dGNR split into two sGNRs at a Y-shaped splitting point. Each plot was obtained by counting over 100 GNRs in the AFM images.

The purity of the GNRs was also analysed by Raman spectroscopy, as shown in Fig. S3. G and D peaks were observed in all the spectra; each spectrum was measured at a different position of the same sample. Since no RBM peaks were observed in any of the spectra, the Raman data supported the conclusions drawn on the basis of the AFM images, that is, the obtained material consisted of highly purified sGNRs.

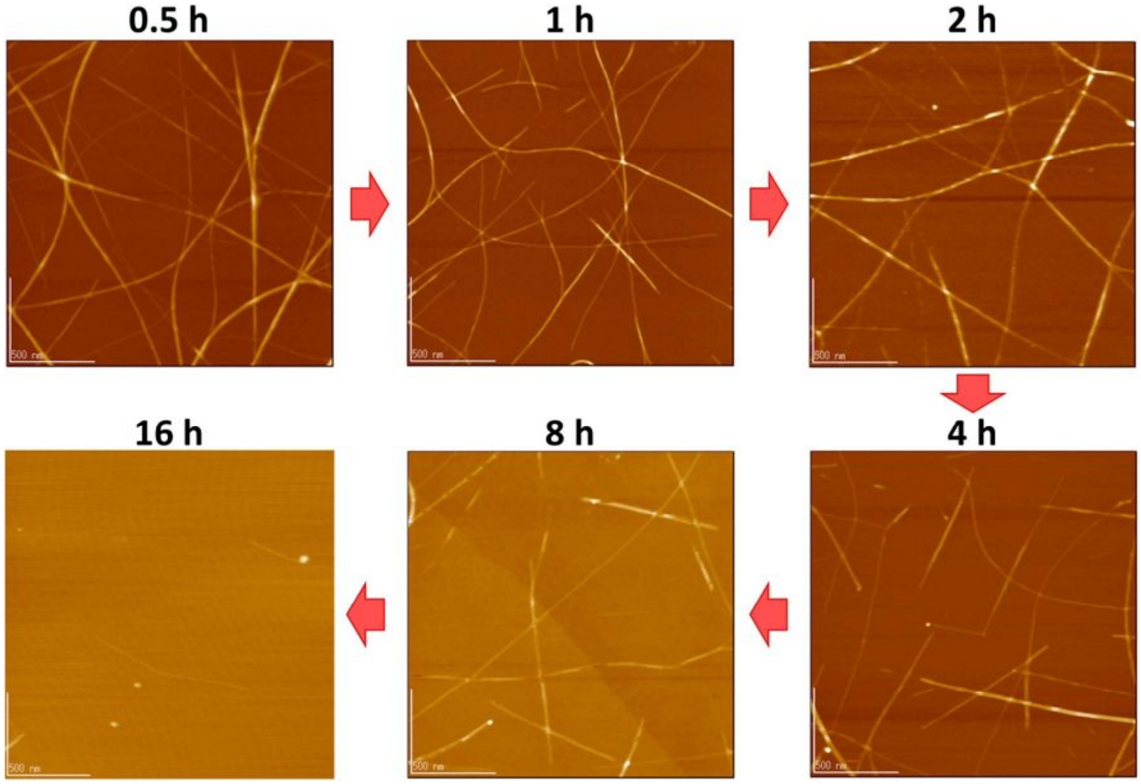

Fig. S2. AFM images of the GNRs obtained for different sonication times.

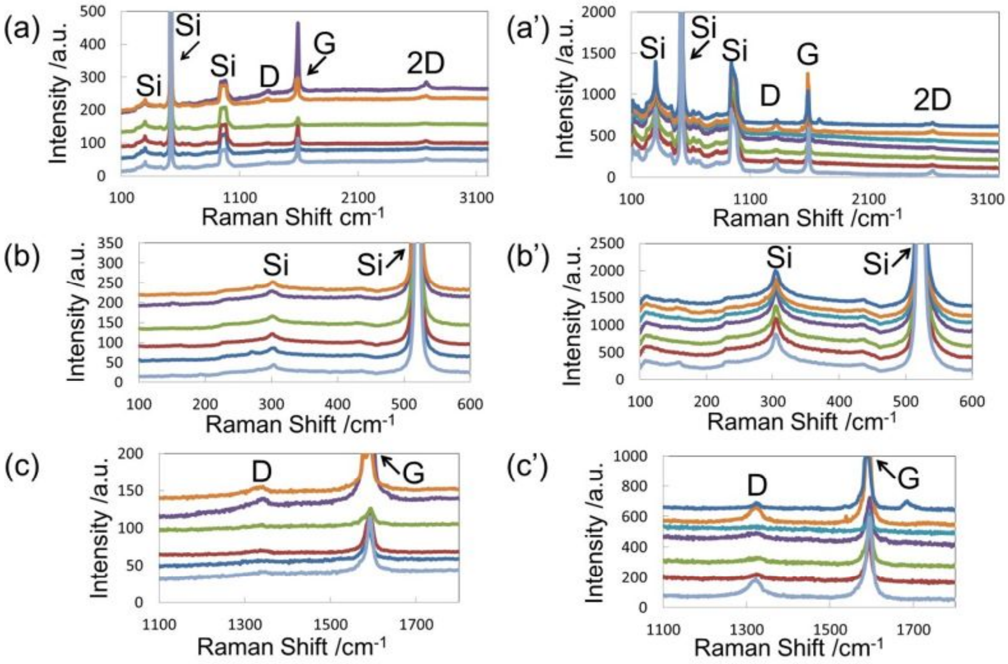

Fig. S3. Raman spectra obtained from the same sample using (a) 532 nm and (a’) 633 nm excitation lasers. Graphs (b) and (b’) are magnified versions of the regions extending from 100 to 600 cm^{-1} in (a) and (a’), respectively. Graphs (c) and (c’) are magnified versions of the regions extending from 1100 to 1800 cm^{-1} in (a) and (a’), respectively. Since peaks attributable to the radial breathing mode (RBM) were not observed, it can be concluded that these data are attributable to highly purified GNRs. The term "a.u." means "Arbitrary Unit."

The results of electrical measurements performed on a 23-nm-wide sGNR and a

29-nm-wide dGNR confirmed that the obtained material was indeed GNRs (Fig. S4). The *I*-*V* curves did not contain a plateau zone, and the d*I*/d*V* curves exhibited a Dirac point at 0 V. This further confirmed that the GNRs were obtained.

The curves for the sGNRs, shown in Fig. S4, were obtained using a three-electrode FET structure. The conductance (d*I*/d*V* at 0 V) of the dGNRs was about twice that of the sGNRs, because of the higher current flow in the case of the former.

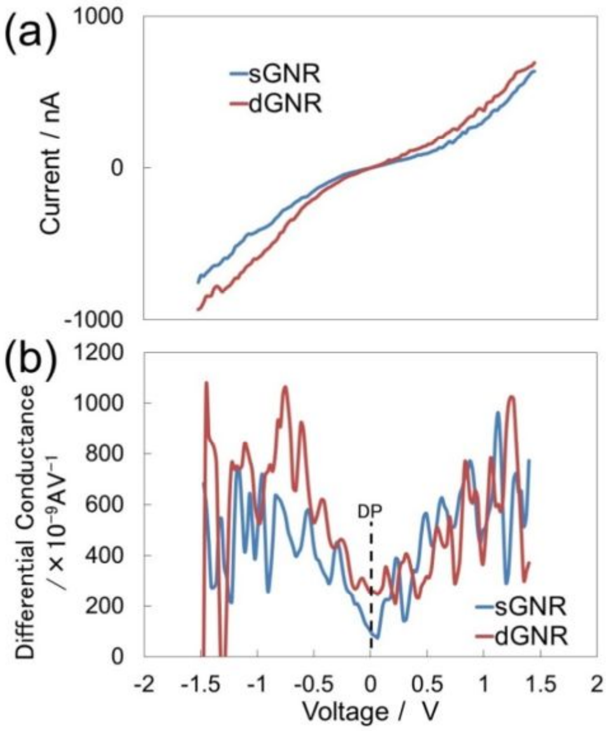

Fig. S4. (a) *I*-*V* and (b) differential conductance (d*I*/d*V*-*V*) curves of the sGNRs and dGNRs. The differential conductance curves of both types of GNRs showed a Dirac point (DP in (b)), corresponding to semimetallic conduction, at approximately 0 V. The widths of the sGNR and dGNR tested were 23 nm and 29 nm, respectively.

The sGNR yields when SWNTs and DWNTs were used as the starting materials were compared as following: the two types of CNTs were used in the same amounts to obtain solutions of sGNRs. Then, similar volumes of these solutions were cast on substrates. As a result, it was possible to count the number of sGNRs in similar-sized areas of the AFM images of the samples. The number of sGNRs obtained from DWNTs was more than double of that obtained from SWNTs. This result implies that the yield of sGNRs when DWNTs were used as the starting material was more than double of that in the case when SWNTs were used as the starting material.
